# Transcriptome analysis reveals differential immune related genes expression in bovine viral diarrhea virus-2 infected goat peripheral blood mononuclear cells (PBMCs)

**DOI:** 10.1186/s12864-019-5830-y

**Published:** 2019-06-21

**Authors:** Wenliang Li, Li Mao, Xin Shu, Runxia Liu, Fei Hao, Jizong Li, Maojun Liu, Leilei Yang, Wenwen Zhang, Min Sun, Chunyan Zhong, Jieyuan Jiang

**Affiliations:** 1 Key Laboratory of Veterinary Biological Engineering and Technology, Ministry of Agriculture, Institute of Veterinary Medicine, Jiangsu Academy of Agricultural Sciences, Nanjing, 210014 People’s Republic of China; 2 School of Food and Biological Engineering, Jiangsu University, Zhenjiang, 212013 People’s Republic of China; 3 College of Veterinary Medicine, Nanjing Agricultural University, Nanjing, 210095 People’s Republic of China; 4 South Dakota State University, Brookings, SD 57007 USA; 5 College of Animal Science, Guizhou University, Guiyang, 550000 People’s Republic of China

**Keywords:** Bovine viral diarrhea virus, RNA-Seq, Transcriptome, Innate immune response, Chemokine, Cytokine

## Abstract

**Background:**

Bovine viral diarrhea virus (BVDV) is an economically important viral pathogen of domestic and wild ruminants. Apart from cattle, small ruminants (goats and sheep) are also the susceptible hosts for BVDV. BVDV infection could interfere both of the innate and adaptive immunity of the host, while the genes and mechanisms responsible for these effects have not yet been fully understood. Peripheral blood mononuclear cells (PBMCs) play a pivotal role in the immune responses to viral infection, and these cells were the target of BVDV infection. In the present study, the transcriptome of goat peripheral blood mononuclear cells (PBMCs) infected with BVDV-2 was explored by using RNA-Seq technology.

**Results:**

Goat PBMCs were successfully infected by BVDV-2, as determined by RT-PCR and quantitative real-time RT-PCR (qRT-PCR). RNA-Seq analysis results at 12 h post-infection (hpi) revealed 499 differentially expressed genes (DEGs, fold-change ≥ ± 2, *p* < 0.05) between infected and mock-infected PBMCs. Of these genes, 97 were up-regulated and the remaining 352 genes were down-regulated. The identified DEGs were found to be significantly enriched for locomotion/ localization, immune response, inflammatory response, defense response, regulation of cytokine production, etc., under GO enrichment analysis. Cytokine-cytokine receptor interaction, TNF signaling pathway, chemokine signaling pathway, etc., were found to be significantly enriched in KEGG pathway database. Protein-protein interaction (PPI) network analysis indicated most of the DEGs related to innate or adaptive immune responses, inflammatory response, and cytokine/chemokine-mediated signaling pathway. TNF, IL-6, IL-10, IL-12B, GM-CSF, ICAM1, EDN1, CCL5, CCL20, CXCL10, CCL2, MAPK11, MAPK13, CSF1R and LRRK1 were located in the core of the network and highly connected with other DGEs.

**Conclusions:**

BVDV-2 infection of goat PBMCs causes the transcription changes of a series of DEGs related to host immune responses, including inflammation, defense response, cell locomotion, cytokine/chemokine-mediated signaling, etc. The results will be useful for exploring and further understanding the host responses to BVDV-2 infection in goats.

**Electronic supplementary material:**

The online version of this article (10.1186/s12864-019-5830-y) contains supplementary material, which is available to authorized users.

## Background

Bovine viral diarrhea virus (BVDV) is the prototypic member of the genus *Pestivirus* in the family *Flaviviridae*, and two main different BVDV species have been recognized as BVDV-1 and BVDV-2 [1]. BVDV infection decreases productive performance and causes considerable economic losses in cattle industry worldwide [2]. Infections with both species of BVDV can induce similar diseases, from subclinical infections to severe clinical diseases including acute diarrhea, respiratory diseases, reproductive failures, congenital defects, and increased mortality due to immunosuppression [2–4]. Persistent infection is the common type of infection in cattle and the persistently infected (PI) animals are considered the main source of BVDV transmission [2, 5]. PI animals had also been detected in heterologous species, which amplify and facilitate the reservoirs for BVDV [6, 7]. Evidence of BVDV infection exists in 7 families (over 50 species) of *Artiodactyla* including *Antilocapridae, Bovidae, Camelidae, Cervidae, Giraffidae, Suidae,* and *Tragulidae* [6]. The circulation of BVDV-1 and BVDV-2 in cattle, pigs had been identified in China [8–10]. Our previous study has identified the prevalence of BVDV-1 in Chinese goat herds [11]. The infection of BVDV-2 in goat or sheep has been confirmed in India, Korea [12–14]. Therefore, the risk and prevalence of BVDV-1 and BVDV-2 in goat/sheep herds needs urgent attention.

Recently, high-throughput RNA-Sequencing (RNA-Seq) technologies give the opportunity to produce large numbers of sequence data in non-model organisms, and this method is better than the traditional microarray analysis [15, 16]. It provides a thorough understanding of the host defense mechanisms and immune evasion strategies of viral infection [17]. The transcriptional landscape in the host upon virus infection facilitates the understanding of host immune responses and defense mechanisms upon the pathogenic microorganism infection at whole mRNA level, and provides new approaches to the potential control of virus infections.

Two biotypes of BVDV are recognized: cytopathic (cp) and non-cytopathic (ncp) strains. In experimental infected calves, BVDV-specific antibody is first detected shortly after viral clearance for both biotypes. T cell proliferative responses are detectable by 3–4 weeks post-infection with cpBVDV; while delayed to about 6–8 weeks after ncpBVDV infection [18]. The primary site of BVDV replication is immune tissue, viral replication results in altered cell function or cell death in different lymphoid populations. The resulting immune suppression occurs in all acute BVDV infections [19]. Peripheral blood mononuclear cells (PBMCs), including lymphocytes, monocytes and macrophages, play a pivotal role in the host innate or adaptive immune responses to viral infection. PBMCs were the main target of BVDV infection, infection of lymphocytes and monocytes by BVDV resulted in lymphoid depletion of B cells, T helper cells, cytotoxic T cells and γ-δ T cells [20, 21]. PBMCs have been proved to be a suitable model for characterizing the host immune responses to virus infection and have been utilized for the evaluation of immune responses to animal viruses [17, 22, 23]. Global transcriptome analysis has been employed to explore the molecular events of host interaction with BVDV in bovine originated cells [24–27]. However, no report on BVDV-goat interactome is available to date. In this study, Illumina sequencing method was used to identify the transcriptome changes in BVDV-2 infected goat PBMCs. For the first time, we obtained the differentially expressed transcriptome profile in the goat PBMCs during BVDV-2 infection. The results will be helpful for better understanding the host responses to BVDV-2 infection and its relationship to viral pathogenesis in goats.

## Results

### Determination of BVDV-2 replication in goat PBMCs

**Fig. 1:**
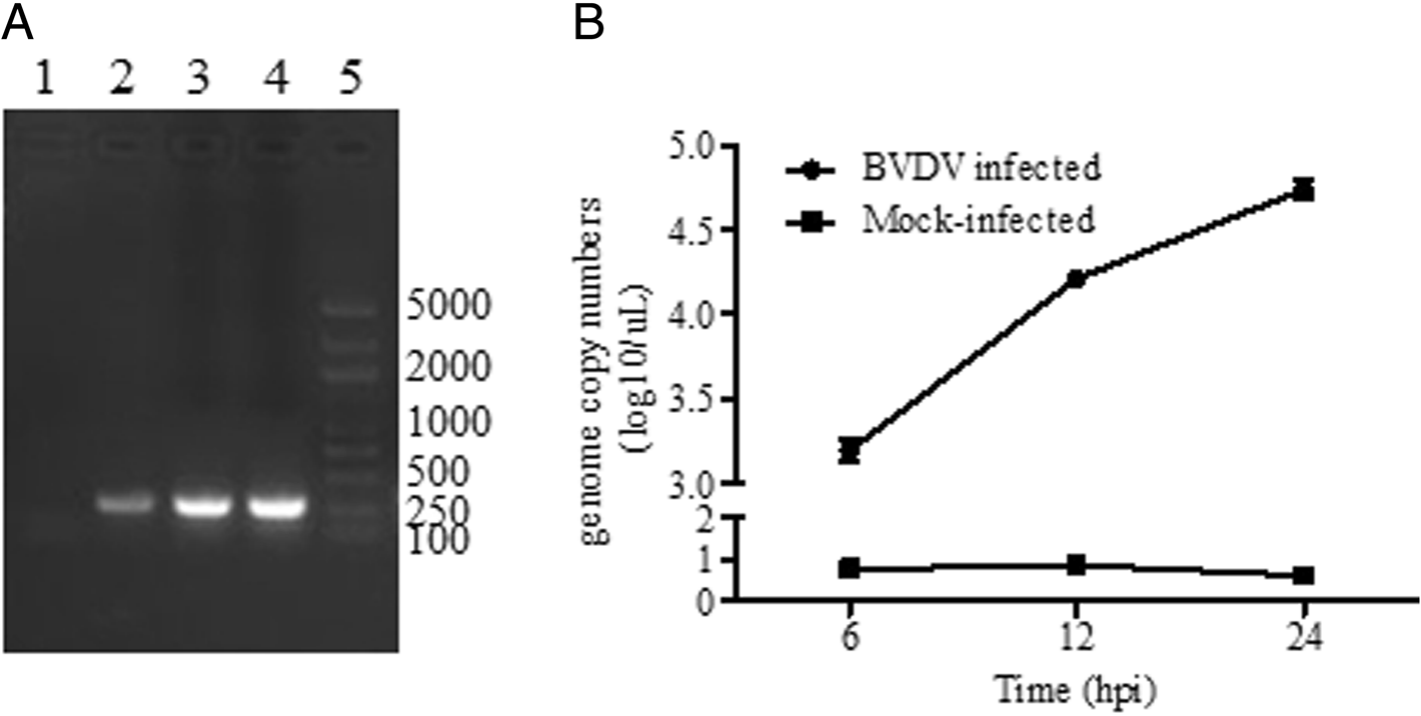
Identification of viral infection in goat PBMCs. **a** Amplification of 5’UTR by RT-PCR at 6 h, 12 h and 24 hpi in BVDV-2 infected goat PBMCs. 1: Mock-infected PBMC at 12hpi; 2: Infected PBMC at 6 hpi; 3: Infected PBMC at 12 hpi; 4: Infected PBMC at 24 hpi; 5: DNA Marker DL-2000 plus. **b** Detection of viral genome copy numbers in goat PBMCs by qRT-PCR at different time points

To confirm the replication of BVDV-2 in goat PBMCs, RT-PCR and qRT-PCR were performed. As shown in Fig. 1a, 5’-UTR fragment with ~ 290 bp was amplified in infected goat PBMCs from 6 to 24 h post- infection (hpi). qRT-PCR detection showed similar results, the BVDV genome copy numbers increased from 6hpi and reached high levels at 12 and 24 hpi (Fig. 1b). These results confirmed the BVDV-2 infection in goat PBMCs. To explore the effect of early BVDV replication on gene expression, 12 hpi was selected for sampling and RNA-Seq. In addition, BVDV nucleotide was not detected in the mock infected PBMCs at any of the experimental time points.

### Transcriptome quantification

After RNA-seq, a total of 122,154,110 raw reads (B2: 62679448; N: 59474662) were obtained. After removing low-quality reads and reads with adaptor sequences, 113,957,630 clean reads (B2: 58748222; N: 55209408) were obtained (Table 1). We then queried the clean reads against the latest reference genome (Gallus_gallus-5.0, https://www.ncbi.nlm.nih.gov/assembly/GCF_001704415.1) and mapped using TopHat (http://tophat.cbcb.umd.edu/). For B2 and N samples, 50,308,617 and 47,349,951 reads were mapped to the reference genome with mapped rate of 85.63 and 85.76%, respectively. Among the matched 13,974 target genes for B2, 9883 with RPKM≥1; For N, 13905 target genes were matched and 9952 with RPKM≥1 (Table 1).

**Table 1:** Summary of reads quality and mapping results of RNA-Seq

Sample	Total raw reads	Total clean reads	Mapped reads	Mapped rate (%)	FPKM> 0	FPKM> 1	FPKM> 5	FPKM> 10
B2	62,679,448	58,748,222	50,308,617	85.63	13,974	9883	7089	5160
N	59,474,662	55,209,408	47,349,951	85.76	13,905	9952	7120	5162

### DEGs analysis and functional annotation

**Fig. 2:**
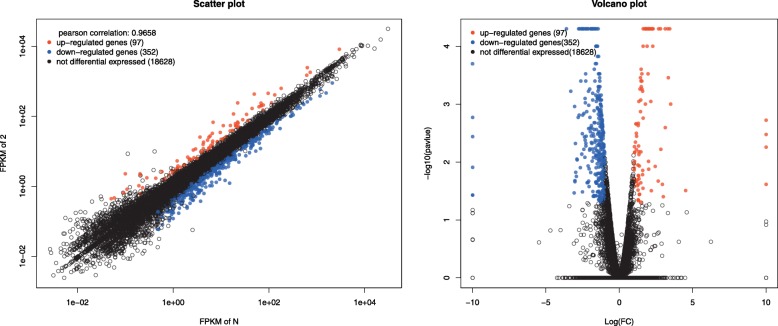
Summary of the differentially expressed genes between mock and BVDV-2 infected samples at 12hpi

After the gene mapping and the Cuffdiff analyses in terms of FRKM, a total of 449 genes were identified as significantly differentially expressed for infected group (B2), when comparing with the mock-infected control group (N, fold change (FC) ≥ ± 2, *p* < 0.05). Among the 449 genes, 97 were up-regulated and 352 genes were down-regulated (Fig. 2, Additional file 4: Figure S1, Additional file 1: Table S1).

**Fig. 3:**
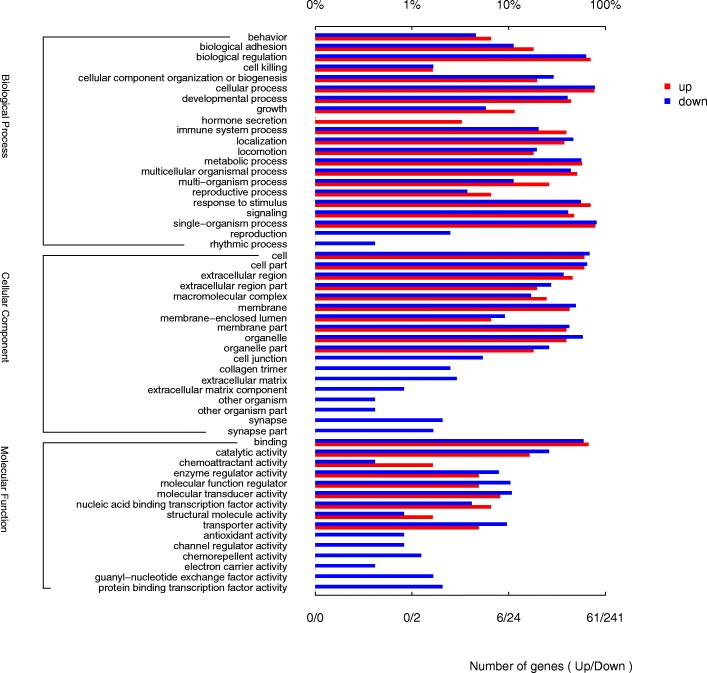
GO annotation for the DEGs between mock and BVDV-2 infected goat PBMCs

The 449 DEGs were annotated to 54 different GO terms. The up-regulated DEGs were annotated to 38 GO terms and the down-regulated DEGs were annotated to 53 GO terms (Fig. 3). The most annotated GO terms were metabolic process (BP), cellular process (BP), response to stimulus (BP), biological regulation (BP), localization (BP), cell (CC), cell part (CC), membrane (CC), membrane part (CC), extracellular region (CC), organelle (CC) and binding (MF), etc. (Fig. 3).

### Functional and PPI analysis of DEGs

**Fig. 4:**
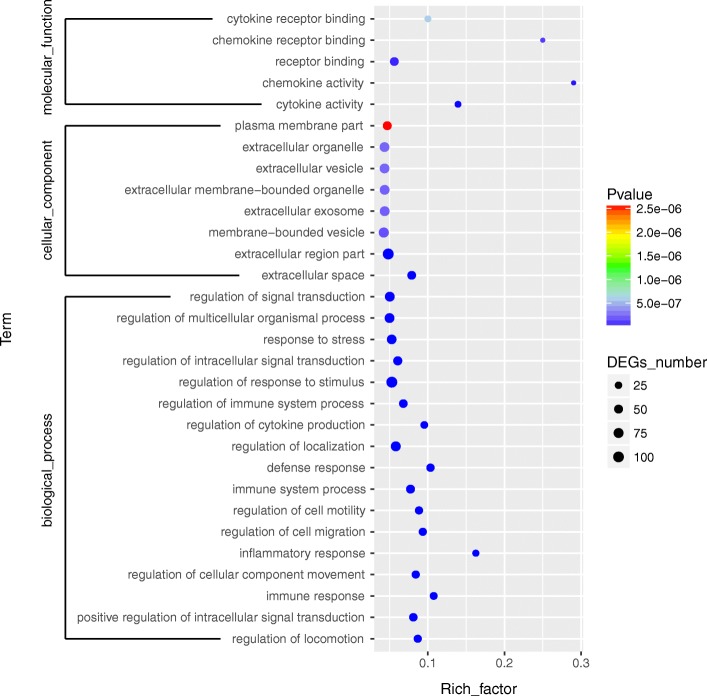
GO enrichment analysis for the DEGs between mock and BVDV-2 infected goat PBMCs. Circles indicate numbers of enriched genes and colors depict the *P* value

After GO enrichment analysis, DEGs were enriched into different GO terms. For immune related DEGs, significant enrichment was observed in regulation of locomotion/ localization, immune response, inflammatory response, immune system process, defense response, regulation of cytokine production of BP group and in cytokine activity, chemokine activity, receptor binding of MF group. (Fig. 4 and Additional file 2: Table S2).

**Fig. 5:**
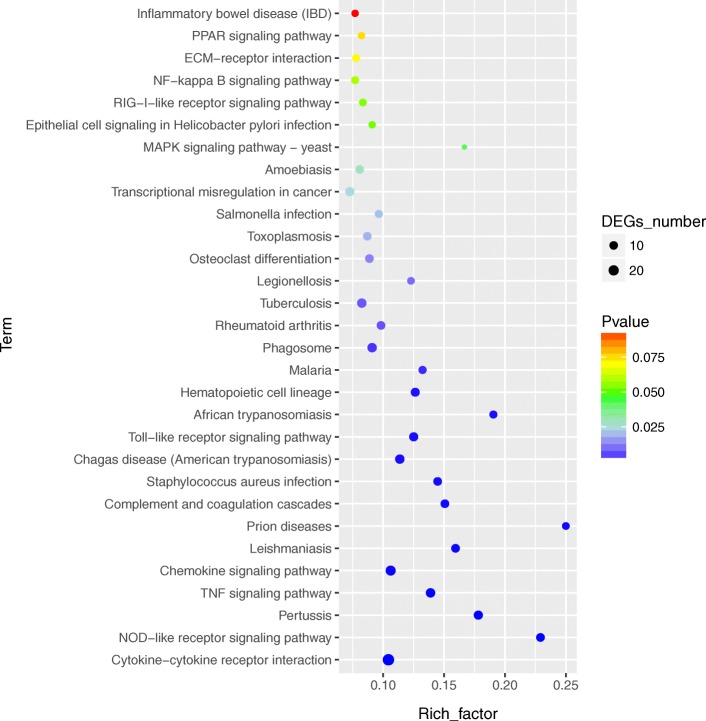
Top 30 pathways enriched in KEGG database for the DEGs between mock and BVDV-2 infected goat PBMCs. Circles indicate numbers of enriched genes and colors depict the *P* value

To further define DEGs function, KEGG pathway/enrichment analysis was performed. Among the fifteen significantly enriched pathways, cytokine-cytokine receptor interaction, TNF signaling pathway, chemokine signaling pathway, complement and coagulation cascades and NOD-like receptor signaling pathway were found to be enriched to canonical pathways (Fig. 5, Table 2 and Additional file 3: Table S3). The cytokine-cytokine receptor interaction pathway was the pathway enriched with most number of DEGs (*n* = 29). For the 29 DEGs, CCL4, CCL3, CXCL10, CCL5, CCL22, CCL20, GM-CSF, TNF, IL-6, IL-17A, IL-12B, IL-19, IL-10, TNFRSF13C, TNFRSF8, TNFRSF9 and XCL1 were up-regulated; while TNFSF12, CSF1R, TNFRSF21, CSF3R, regakine-1(LOC102170772), CCL2, CCL24, CCL17, CCL14, CCL25, IL-5RA and PPBP were down-regulated. In addition, TNF, IL-6, CXCL10, CCL4, CCL3, CCL5, CCL20, CCL2 and regakine-1 were enriched in at least three of the six pathways mentioned above (Table 2).

**Table 2:** Lists of the significantly enriched KEGG pathways associated with immune responses

Pathways	ID	DEGs No.	p-value	Up-regulated genes	Down-regulated genes
Cytokine-cytokine receptor interaction	ko04060	29	5.04E-06	CCL4,CCL3,CXCL10,IL17A,IL12B,IL19,IL10, XCL1,CCL5,CCL22,IL6,CCL20,GM-CSF,TNF, TNFRSF13C,TNFRSF8, TNFRSF9	TNFSF12,CSF1R,regakine-1,CCL2,TNFRSF21, CSF3R,CCL24,CCL17,CCL14,IL5RA,CCL25, PPBP
NOD-like receptor signaling pathway	ko04621	11	1.01E-05	TNFAIP3,NFKBIA,IL6,TNF,CCL5	CARD9,MAPK11,NLRP1,CCL2,MAPK13,NOD1
TNF signaling pathway	ko04668	15	5.59E-05	TNF,NFKBIA,CCL5,IL6,TNFAIP3,EDN1,PTGS2,CCL20,ICAM1,GM-CSF,CXCL10	MAPK11,CREB3L2,CCL2,MAPK13
Chemokine signaling pathway	ko04062	19	0.000168	CCL3,CCL20,CXCL10,CCL4,CCL5,CCL22, XCL1,NFKBIA, GNG7	CCL17,CCL24,PAK1,CCL25,regakine-1,CCL2,CCL14,GNB4,GNG12,PPBP
Complement and coagulation cascades	ko04610	11	0.000286	C3,SERPING1,CR2	C1QA, C1QB,C1QC, CFD, F13A1,C5AR1, CD55, LOC102185401
Toll-like receptor signaling pathway	ko04620	13	0.000434	IL12B,CCL3,CXCL10,TNF,NFKBIA,CCL4, CCL5,IL6	CD14,MAPK11,CD86,regakine-1,MAPK13

**Fig. 6:**
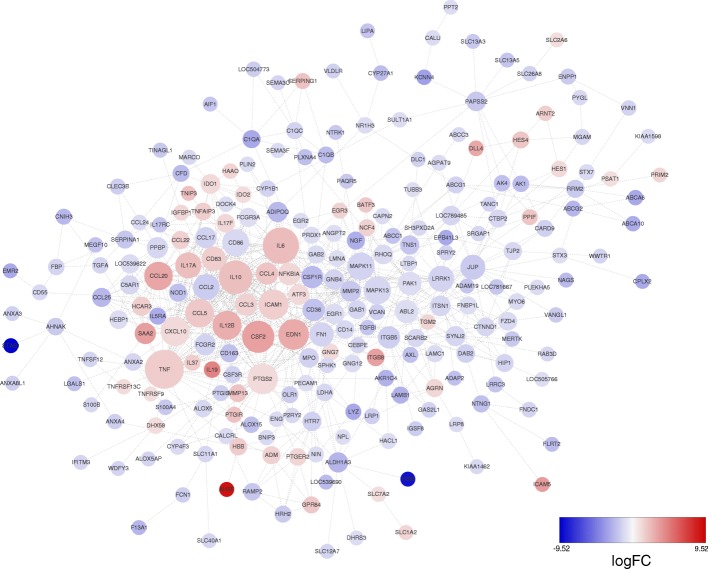
PPI network of the selected DEGs based on STING analysis results and fold change information. The up-regulated genes are shown in red and down-regulated genes are shown in blue with the gradient showing the extent of expression. The size of the node indicates connectivity

STRING analysis was used to explore the potential interaction network of the DEGs. As shown in Fig. 6, most of the DEGs related to innate or adaptive immune responses, inflammatory response, cytokine/chemokine-mediated signaling pathway, etc. Among the up-regulated genes, TNF, IL-6, IL-10, IL-12B, GM-CSF, ICAM1, EDN1, CCL20, CXCL10 and CCL5 were located in the core of the network and linked to lots of other DGEs; for the down-regulated genes, the key points included CCL2, MAPK11, MAPK13, CSF1R and LRRK1, etc., which linked to more genes. In addition, not all DEGs showed connection with others because their functions were either unrelated or have not yet been clarified (Additional file 5: Figure S2). These DGEs were not included in Fig. 6 during the analysis.

### Partial validation of RNA-Seq data

**Fig. 7:**
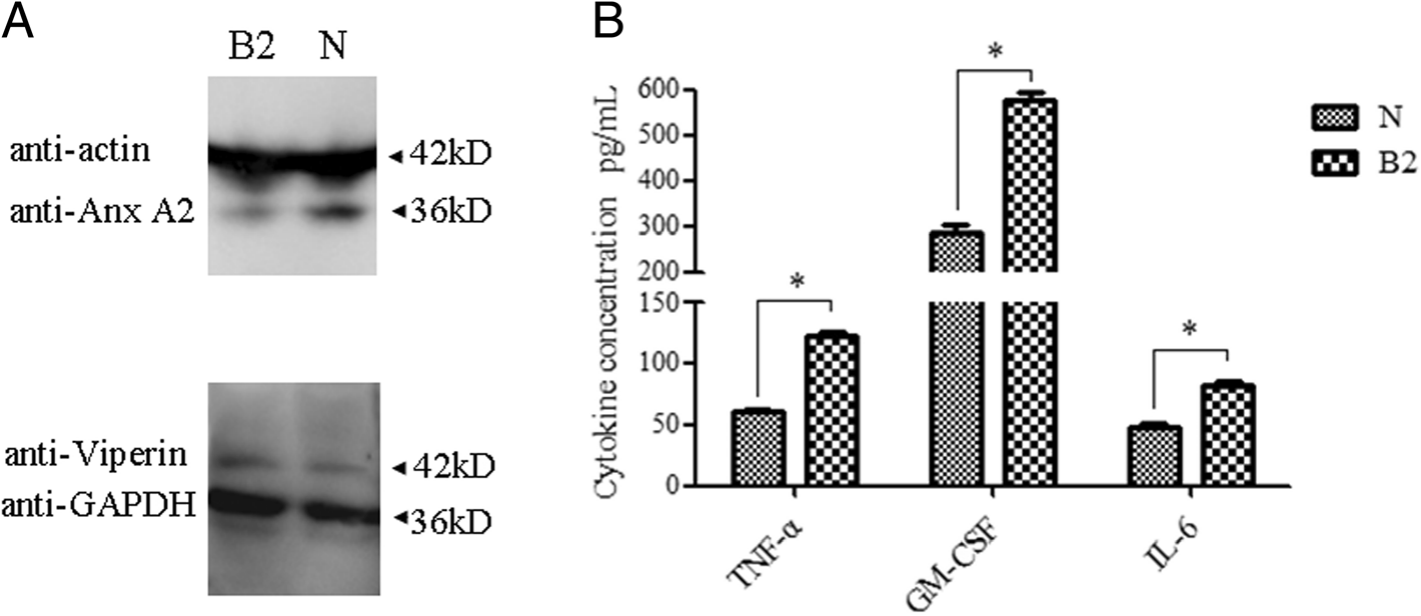
Partial validation of RNA-Seq data by Western blot and ELISA. **a** The expression of Annexin A2 and Viperin in the samples was determined by Western blot using rabbit anti-Viperin polyclonal antibody (Abcam) and mouse anti-AnnexinA2 antibody (Santa Cruz). **b** The concentration of GM-CSF, TNF- and IL-6 present in the samples were determined by commercial ELISA kit and calculated with the formula derived from the standard curve. Data was shown as the mean ± S.D. Columns marked with * (P < 0.05) are significantly different from each other

To further validate the RNA-Seq data, DEGs with annotations associated with immune responses from RNA-seq were selected for qRT-PCR analysis. As shown in Table 3, eighteen selected genes exhibited a concordant direction both in RNA-Seq and qRT-PCR analysis. The correlation coefficient between RNA-Seq and qRT-PCR results was high (R^2^ = 0.91). Some of the DEGs were further determined by Western blot or ELISA, as shown in Fig. 7, the expression of Annexin A2 decreased obviously (Fig. 7a) and the expression of TNF-α, GM-CSF and IL-6 were increased significantly (Fig. 7b). In addition, Viperin (used as a control) expression showed no change between infected and mock-infected group, which was consistent with the RNA-Seq result. These results confirmed that the differential expression genes identified by RNA-Seq is reliable.

**Table 3:** The immune responses related DEGs and partial validation of RNA sequencing data by qRT-PCR

Gene ID	Gene name	Function annotation	FC log2(B2/N)	
			RNA-Seq	qRT-PCR
Down-regulated				
NC_030809.1_gene90	C1QA	complement C1q subcomponent subunit A	−3.03	
NC_030809.1_gene93	C1QB	complement C1q subcomponent subunit B	−2.29	
NC_030809.1_gene91	C1QC	complement C1q subcomponent subunit C	−1.42	
NC_030814.1_gene546	CFD	complement factor D	−2.35	
NC_030812.1_gene428	S-LE	lysozyme	−3.03	
NC_030836.1_gene709	IFITM3	interferon-induced transmembrane protein 3	−1.36	
NC_030834.1_gene266	LOC102188015	beta-defensin 103A	−1.05	−0.5
NC_030810.1_gene1245	S100A4	protein S100-A4	−1.76	
NC_030808.1_gene1009	S100B	protein S100-B	−1.23	
NC_030818.1_gene1111	FCN1	ficolin-1	−1.56	
NC_030818.1_gene586	ANXA4	annexin A4	−1.54	−0.7
NC_030813.1_gene593	ANXA3	annexin A3	−1.49	−0.9
NC_030817.1_gene452	ANXA2	annexin A2	−1.29	−1.9
NC_030826.1_gene194	CCL2	C-C motif chemokine 2	−1.61	−2.2
Up-regulated				
NC_030828.1_gene424	NFKBIA	NF-kappa-B inhibitor alpha	1.27	1.3
NC_030836.1_gene157	LOC102168428	serum amyloid A protein	1.31	2.2
NC_030836.1_gene161	LOC100860781	serum amyloid A3	1.62	
NC_030826.1_gene156	CCL3	C-C motif chemokine 3	1.48	1.5
NC_030825.1_gene308	CCL22	C-C motif chemokine 22	1.48	
NC_030826.1_gene155	CCL4	C-C motif chemokine 4	1.95	1.8
NC_030826.1_gene163	CCL5	C-C motif chemokine 5	1.80	1.6
NC_030809.1_gene360	CCL20	C-C motif chemokine 20	3.12	2.7
NC_030813.1_gene567	CXCL10	C-X-C motif chemokine 10	1.16	1.2
NC_030814.1_gene1059	ICAM1	intercellular adhesion molecule 1	1.54	
NC_030830.1_gene594	TNF	tumor necrosis factor alpha	1.62	3
NC_030823.1_gene84	IL-10	interleukin-10	2.16	2.5
NC_030830.1_gene686	IL-17A	interleukin-17A	2.18	1.6
NC_030811.1_gene808	IL-6	interleukin-6	2.28	2.4
NC_030814.1_gene174	IL-12B	interleukin-12 subunit beta	2.80	2.2
NC_030814.1_gene828	GM-CSF	granulocyte-macrophage colony-stimulating factor	3.30	2.45

## Discussions

BVDV is one of the most important viral diseases of various species of domestic animals. Regardless of clinical presentation, all BVDV infections result in significant loss of immune tissue, the resulting immune suppression and increased severity of subsequent infections [28]. The different genotypes and biotypes of BVDV, wide spectrum of susceptible host, ability to induce persistently infection, as well as its ability to interfere both innate and adaptive immunity of the host, make it difficult for prevention and control.

Microarray and RNA-Seq analysis are the two main techniques for transcriptome analysis. There were many reports about transcriptome changes upon animal virus infection by microarray analysis [29–31]. As a revolutionary tool, RNA-Seq has been widely used in recent years, such as BTV, PRRSV, PPRV and NDV [22, 32–34]. Several studies had explored the effect of BVDV infection on mRNA expression changes in different bovine cells [25, 27, 35], but understanding of BVDV-host interaction is still far from complete. Comparing to the studies in cattle little is known regarding the cellular impact of BVDV-2 infection in goats. Herein, in the present study, goat PBMCs were infected with a ncp BVDV-2 strain, and the transcriptome was evaluated at 12 hpi to identify the global gene expression changes, to understand and delineate the mechanism of early responses induced by virus infection. Transcriptional changes of the DEGs involved in immunological processes were analyzed specially. The results from GO, KEGG and PPI analysis indicated that various numbers of DEGs were involved in different biological processes of host immune responses.

The innate immunity is the first defense line against infectious disease. The PBMCs were the gatekeeper of virus spread during systemic viral infections and include several subpopulations that may cooperate in the activation processes [36]. These cells play important role in innate and adaptive immune responses. The pathogen-associated molecular pattern receptors (PAMPs) such as Toll-like receptors (TLRs) or RIG-I-like receptors (RLRs) recognize pathogens, then released a series of related antiviral cytokines. In this study, under BVDV-2 infection, the expression of TLRs, RLRs, interferon (IFN) and the ISGs were not significant induced. Contrarily, the mRNA levels of lysozyme, beta-defense, competent of complement system (C1q, C1r, C1s, CFD) and IFITM3 (one of the ISGs) was found to be down-regulated. A previous study showed that TLR3, type I IFN gene was up-regulated in ncp BVDV-infected monocytes at 1hpi but not at 24hpi [37]. It has also been reported that ncpBVDV dose not induce type I IFN in vitro and block the induction of type I IFN by dsRNA or other viruses and interferon tau-stimulated ISGs expression [18, 38]. The interaction of ncp BVDV with its host cells impairs both of the innate and adaptive immunity [39]. During BVDV infection, viral RNA firstly triggers IFN synthesis, while the viral RNase E^rns^ protein inhibits IFN expression; in addition, N^pro^ promotes the degradation of IRF-3, which effectively blocks IFN expression in BVDV-infected cells [39–41]. So, the inhibition of immune genes mentioned above might result from the interference of BVDV-2 infection on the related immune pathways. The inhibition of IFN synthesis and other competent of innate immunity might play an important role in escaping innate immunity and the establishment of effective infection for BVDV in host cells.

The complement system, which consists of both soluble factors and cell surface receptors, is one of the major innate defense systems. The main role of complement system is to protect against infections, it also links the innate and adaptive immune responses [42]. In the present study, C1q, C1r, C1s, CFD were down-regulated, while C3 was up-regulated; imply that BVDV-2 infection in goat PBMCs may inhibit the classical or alternative pathways of the complement system activation.

Inflammation was one of the important anti-microorganism responses upon infection. Once exposed to infectious agents, host cells produced certain cytokines, such as TNF-α, IL-1 and IL-6, resulting in the development of inflammation. TNF-α is an essential mediator of inflammation and also facilitates the transition from innate to adaptive immunity. IL-6 affects both inflammation and adaptive immunity. It promotes some aspects of inflammation, especially in response to tissue damage and severe infections since it is a major mediator of the acute phase reaction and of septic shock. Many viruses, including BVDV, could induce inflammation after infection. Studies in cattle and sheep have shown that the pathology of BVDV associated with the replication of virus and the production of pro-inflammatory cytokines and induced inflammatory responses [43–45]. One study examined the mRNA expression in tracheo-bronchial lymph nodes of BVDV infected beef calves and found that high virulence BVDV-2 strain induced pro-inflammatory (TNF-α, IL-12, IL-1β, IL-2, IFN-γ) and anti-inflammatory (IL-4 and IL-10) cytokines, while low virulence BVDV-1a strain only up-regulated IL-12 and IL- 15 gene expression [46]. In an other study, both cp and ncp BVDV biotypes suppressed pro-inflammatory cytokines TNF-α, IL-1β, and IL-6, but did not change IL-12 and INF-γ gene expression in bovine PBMCs [37]. As a member of pestivirus, virulent CSFV infection in pigs resulted in the secretion of cytokines associated with inflammation or apoptosis such as TNF-α, IL-2, IL-4, IL-6, and IL-10 [47]. The BVDV-2 strain used in this study induced fever, viremia and lymphopenia in experimentally infected goats (unpublished data). The clinical signs, together with the pattern of increased TNF-α, IL-6, IL-12, IL-10 and IL-17 mRNA level observed in BVDV2-infected goat PBMCs in this study, suggested that BVDV-2 induced an acute inflammatory response in the early stage of infection. The enriched pathways related to cytokine/chemokine signaling provide obvious evidence that specific immune responses are activated during that acute phase of BVDV infection.

The serum amyloid A (SAA) family comprises a number of apolipoproteins, which include acute-phase SAAs (A-SAAs) and constitutive SAAs (C-SAAs). A-SAAs have been identified in all vertebrates and could be induced as much as 1000-fold during inflammation [48]*.* Transcription of SAAs members LOC102168428 (SAA like) and LOC100860781 (SAA3) were found to be up-regulated in this study. Members of A-SAAs have been identified to be activated upon infection and proven useful as an inflammatory marker for many viral diseases of animals [49–51]. Similarly, Ganheim C. et al. reported a significant acute phase response with elevated values of SAA and other acute phase proteins in calves experimentally infected with BVDV [52]. So, SAA might be considered as a diagnostic marker for BVDV induced inflammation.

Viral infection usually induces the recruitment of inflammatory cells to the infection site, and this activity is regulated by various cytokines and chemokines. Chemokines are a great superfamily of at least 50 small (8- to 10-kDa) structurally related chemoattractant proteins, which play a key role in initiating the innate and subsequently adaptive immune responses. Chemokines have many roles in the regulation of leukocyte development, angiogenesis, tumor growth and metastasis [53]. They coordinate the migration of leukocytes and hence dictate the course of many inflammatory and immune responses by recruiting different immune cells toward sites of infection [54]. Monocytes/macrophages are part of the first line of defense and have been shown to release a variety of chemokines in response to infection [31]. Studies have determined chemokines expression upon the infection of several viruses, such as PRRSV, PEDV and PCV2 [30, 32, 55]. Study by Helal et al. showed that low expression of CXCR4 and high expression of IL-10 is associated with the production of PI calves in a herd level [56]. Trzeciak-Ryczek A. et al. reported that ncpBVDV infection increases CXCR4, CXCL12 mRNA expression in bovine PBMCs [57]. In another study, detection of chemokine profile in cp and ncp BVDV infected bovine monocytes/macrophages demonstrated up-regulation of several key chemokines of the CCL and CXCL families to cpBVDV, but not ncpBVDV [31]. In our study, BVDV-2 infected goat PBMCs showed increased transcription of CCL3, CCL4, CCL5, CCL20, CXCL10 and decrease of CCL2, suggesting that such chemokines may contribute to the recruitment and regulation of macrophages or other inflammatory cells to the infected sites after infection.

GM-CSF was another up-regulated DEG found in our study. It is not only an inducer of differentiation and proliferation of granulocytes/macrophages but also involved in a wide range of biological processes in both innate and adaptive immunity [58]. GM-CSF has been widely used as adjuvant for vaccines and has been shown powerful as an important therapeutic target in several autoimmune and inflammatory diseases [58, 59]. The induction of GM-CSF expression has been identified in several viruses and shown to alter pathogenic “M1-like” macrophage inflammation after influenza A virus infection [60–62]. The high expression of GM-CSF in goat PBMCs indicated the regulation role of it in host immune responses after BVDV-2 infection.

## Conclusions

In conclusion, the changes of a series of immunity related genes in BVDV-2 infected goat PBMCs were observed by RNA-Seq analysis. The results will be useful for exploring and further understanding host responses to BVDV-2 infection in goats.

## Methods

### Virus and cells

MDBK cells were (purchased from the China Institute of Veterinary Drug Control) cultured in DMEM (Hyclone, USA) supplemented with 10% fetal bovine serum (FBS, Transgen, Bio, Inc.,China) and kept at 37 °C. For the production of stock virus, MDBK cell monolayer was inoculated with the ncpBVDV2 strain C201604 (GenBank No. MG420995, identified and kept in our lab) with multiplicity of infection (MOI) of 0.1. At 3 days post infection, the infected cells were harvested, lysed by three cycles of freeze-thaw, centrifuged at 8000×g and 4 °C for 10 min and then aliquoted and stored at − 70 °C until use.

### Goat, PBMCs culture and virus infection

Goats used for blood collection were purchased from a goat farm (private farm, 210 goats) located in Jurong county, Jiangsu province. The goats were vaccinated against goat pox virus, foot and mouth disease virus, peste des petits ruminants virus and Mycoplasma mycoides subsp.capri. The animal was further screened for BVDV and viral specific neutralizing antibodies using RT-PCR and virus neutralization test, respectively. EDTA anticoagulant whole blood were collected from the goats (*n* = 3) and PBMCs were separated using Histopaque-1077 (Sigma) by density gradient centrifugation at 500×g for 20 min, then washed three times with RPMI-1640 medium at 500×g for 10 min. Cells from each animal were suspended to 1× 10^6^ cells/mL with complete RPMI 1640 medium (RPMI 1640 containing 10% FBS) and seeded in six well plates (*n* = 4, 8 wells/animal). Two plates were infected with BVDV2 (2 × 10^5^ TCID_50_/mL, MOI = 0.1) and the other two plates were served as mock-infected control. Samples from one infected plate and one mock-infected plate were collected at 6, 12 and 24 hpi for virus replication detection. At 12 hpi, medium was discarded and the cells of the left two plates (infected and mock-infected) were washed twice with PBS, mixed and pelleted for RNA-Seq.

### RNA extraction, library preparation and RNA sequencing (RNA-seq)

Total RNA was extracted from the infected (B2) and mock-infected (N) PBMCs using TRIzol reagent (Invitrogen) according the manufacturer’s instructions and genomic DNA was removed using DNase I (TaKara). RNA quality (RNA integrity number, RIN) was determined using Agilent 2100 Bioanalyser and quantified using the ND-2000 (NanoDrop). High-quality RNA sample (OD260/280 = 1.8~2.2, OD260/230 ≥ 2.0, RIN ≥ 6.5, 28S:18S ≥ 1.0, > 10 μg) was used to construct sequencing library.

RNA-Seq transcriptome libraries were prepared by TruSeqTM RNA sample preparation Kit from Illumina (San Diego, CA), using 1 μg of total RNA. Shortly, mRNA was isolated with polyA selection by magnetic oligo (dT) beads and fragmented into small pieces using fragmentation buffer. cDNA synthesis, end repair, A-base addition and ligation of the Illumina-indexed adaptors were performed according to Illumina’s protocol. Libraries were then selected for cDNA target fragments of 200–300 bp on 2% Low Range Ultra Agarose followed by PCR amplified for 15 cycles using Phusion DNA polymerase (NEB). After quantified by TBS380, paired-end libraries prepared for both the infected and mock-infected samples (B2 and N) were sequenced with the Illumina HiSeq PE 2X151bp read length. RNA-Seq was performed by Shanghai Biozeron Biotech Co. Ltd.

### Reads quality control and mapping

The raw paired end reads were trimmed and quality controlled by Trimmomatic with default parameters (http://www.usadellab.org/cms/index.php?page=trimmomatic). Clean reads were then separately aligned to the reference caprine genome (https://www.ncbi.nlm.nih.gov/assembly/GCF_001704415.1) with orientation mode using TopHat software (http://tophat.cbcb.umd.edu/), which can align RNA-Seq reads to a genome in order to identify gene expression and exon-exon splice junctions. It is built on the ultrafast short read mapping program Bowtie2 to map with default parameters.

### Differential expression genes (DEGs) analysis and annotation

To identify DEGs between the two samples, the expression level for each transcript was calculated using the fragments per kilobase of exon per million mapped reads (FPKM) method. Cuffdiff (http://cufflinks.cbcb.umd.edu/) was used for differential expression analysis and the DEGs were selected using the following criteria: the logarithmic of fold change was greater than 2 and the p-fdr should be less than 0.05. All DEGs were subjected to Gene Ontology (GO) annotation based on GO database.

### Functional and protein-protein interaction (PPI) analysis of DEGs

To understand the functions of the DEGs, GO functional enrichment and Kyoto Encyclopedia of Genes and Genomes (KEGG) pathway analysis were carried out by Goatools (https://pypi.org/project/goatools/) and KOBAS (http://kobas.cbi.pku.edu.cn/). DEGs were significantly enriched in GO terms and KEGG pathways when their *p*-value was less than 0.05.

PPI network among the DEGs was analyzed using the STRING (http://string-db.org/) database, which included direct and indirect associations of proteins. After analyzing the result from STRING analysis and expression change information for each DEG, the network figure was drawn for the selected DEGs (connected with one or more DEGs) by using Cytoscape software.

### RT-PCR and quantitative real-time RT-PCR (qRT-PCR)

Total RNAs were extracted from infected and mock-infected PBMCs using TransZol UP reagent (Transgen, Bio, Inc.,China) according to the manufacturer’s instruction.

The RT-PCR was performed to identify the replication of BVDV and carried out with EasyScript one-step RT-PCR supermix (Transgen, Bio, Inc., China) in a 20 μl reaction mixture containing 10 μl of 2 × R-Mix, 20 pM of each primer (F: 5'-ATGCCCWTAGTAGGACTAGCA-3', R: 5'-TCAACTCCATGTGCCATGTAC-3'), 0.4 μl of E-Mix, 2 μl extracted RNA and 6.6 μl ddH_2_O. The reaction was run in a thermocycler (Mjmini, BIO-RAD) with the following program: reverse transcription at 45 °C for 30 min; denaturation at 94 °C for 5 min, 35 cycles composed of denaturation at 94 °C for 30 s, annealing for 30 s at 54 °C and extension at 72 °C for 30 s; and was terminated with a final extension of 10 min at 72 °C. Amplification products were detected by electrophoresis in 1.2% agarose gels.

The qRT-PCR amplification was carried out with TransScript one-step qRT- PCR supermix (Transgen, Bio, Inc., China) in a 20 μl reaction mixture containing 10 μl of 2 × Supermix, 20 pM of each primer (Table 4), 0.5 μl of E-Mix, 0.4 μl of passive reference Dye and 2 μl extracted RNA. The reaction was run in ABI Step One instrument as the following procedure: samples were incubated at 45 °C for 5 min firstly; then heated at 94 °C for 30 s and a two-step cycle (5 s at 94 °C, 30 s at 60 °C) was repeated for 40 cycles. GAPDH was used as the internal control and relative quantification of target gene expression was the target transcript in infected group to that of mock-infected group and expressed as –ΔΔCt.

**Table 4:** Primers used for qRT-PCR

Gene Name	Forward primer 5′-3′	Reverse primer 5′-3′
TNF	TCGTATGCCAATGCCCTCA	GATGAGGTAAAGCCCGTCAGT
GM-CSF	GACACTGCTGCTGTGATGAA	CCCTGCTTGTACAGCTCCA
IL-6	TGGATGCTTCCAATCTGGGT	CTGCTCTGCAACTCCATGAC
IL-10	ATGGGCCTGACATCAAGGAG	ACTCTCTTCACCTGCTCCAC
IL-12B	AAACCAGACCCACCCAAGAA	TGAGGTTTGGTCCGTGAAGA
IL-17A	TCTGAGTCTGGTGGCTCTTG	TGGAGTTCGTGTTCCGGTTA
CCL2	CGCTCAGCCAGATGCAATTA	GTCCTGGACCCATTTCAGGT
CCL3	CCTGCTGCTTCTCCTATGC	TGGAAGATGACACCAGGCTT
CCL4	TCCTCGCAGCTTTGTGATTG	TCAGTTCGAGGTCATCCATGT
CCL5	CCATGGCAGCAGTTGTCTTT	CACCCACTTCTTCTCTGGGT
CCL20	CTCCTGGCTGCTTTGATGTC	ATGTCACAGGCTTCATTGGC
CXCL10	ATACACGCTGTACCTGCATC	TGTGGCAATAATCTCGACACG
SAA	ATCACAGACCCTCTGCTCAAG	CCATTCGTTGGCAAACTGGT
NFKBIA	CCTTCAGACACTGCCAGAGA	CTCCAAGCACACAGTCATCG
Beta-Defense	ACCTTCTCTTTGCGTTGCTC	CGTAACCCGCTTATGATGCC
ANXA2	CACACCTCCAAGTGCATACG	ACCTCATCCACACCTTTGGT
ANXA3	AACGGCAGCTGATTGCTAAG	GGCTACCATGAGACCCTTGA
ANXA4	TGAGGGCTGCTTGATTGAGA	GCCCATATTGCAGCTGGTAG
GAPDH	ATGATTCCACCCACGGCAA	ATCACCCCACTTGATGTTGGC

### Virus titration

For titration of virus stock, four replicates of 10-fold serially diluted virus (starting from 1/10) were inoculated on MDBK cell monolayer in 96-well culture plates. After 48 h incubation, the culture plates were fixed at 4 °C for 30 min with ice cold absolute ethyl alcohol and subjected to immunofluorescence staining with BVDV-specific mouse monoclonal antibody Mix (RAE2020, AHVLA, UK; 1:200 diluted in PBS) and FITC-conjugated goat anti-mouse IgG (BOSTER, Wuhan, China; 1:200 diluted in PBS). The fluorescence signals were observed undera fluorescence microscopy (ZEISS) and viral titers was expressed as the 50% tissue culture infective dose (TCID_50_)/mL by Reed-Muench method.

### Western blot

Cell lysates samples were separated by 12% SDS-PAGE and transferred onto nitrocellulose membranes (Pall) using a semi-dry transfer cell (Bio-Rad) at 1 V/cm2 for 40 min. The membrane was treated sequentially with 5% skimmed milk in PBST (PBS containing 0.05% Tween-20) at 37 °C for 2 h, with different primary antibodies (1/200 diluted rabbit anti-Viperin polyclonal antibody (Abcam), 1/400 diluted mouse anti-AnnexinA2 antibody (Santa Cruz), 1/1000 diluted anti-β-actin/GAPDH monoclonal antibody (Transgen, Bio, Inc., China)) at 37 °C for 2 h, and with different secondary antibodies (1/1000 diluted rabbit anti-mouse or goat anti-rabbit IgG antibody conjugated to HRP (Transgen, Bio, Inc., China)). After three washes with PBST, the color development was performed using enhanced chemiluminescence luminal reagent (Thermo Scientific Pierce).

### Elisa

The concentration of GM-CSF, TNF-α and IL-6 present in the infected and mock-infected PBMCs samples were determined by commercial ELISA kit (Jiangsu Yutong Biotech Co., Ltd., China) according to the manufacturer’s instructions and calculated with the formula derived from the standard curve.

## Additional files

Additional file 1: **Table S1.** Differentially expressed genes between mock and BVDV-2 infected samples at 12hpi. (XLS 111 kb)

Additional file 2: **Table S2.** GO enrichment results of the DEGs between mock and BVDV-2 infected samples. (XLS 159 kb)

Additional file 3: **Table S3.** KEGG pathway enrichment result of the DEGs between mock and BVDV-2 infected samples. (XLS 17 kb)

Additional file 4: **Figure S1.** Heat map of the DEGs between mock and BVDV-2 infected samples. Red indicates up-regulation and blue indicates down-regulation. (PDF 47 kb)

Additional file 5: **Figure S2.** STRING analysis of all DEGs between mock and BVDV-2 infected samples. (SVG 8290 kb)

## Data Availability

The datasets used and/or analyzed during the current study available from the corresponding author on reasonable request.

## Abbreviations

BVDV: Bovine viral diarrhea virus
DEGs: Differentially expressed genes
FPKM: Fragments per kilobase of exon per million mapped reads
GO: Gene Ontology
Hpi: Hours post infection
IFN: Interferon
ISGs: Interferon stimulated genes
KEGG: Kyoto Encyclopedia of Genes and Genomes
MOI: Multiplicity of infection
PAMPs: Pathogen-associated molecular pattern receptors
PBMCs: Peripheral blood mononuclear cells
PBST: PBS containing 0.05% Tween-20
RLRs: RIG-I-like receptors
RNA-Seq: RNA sequencing
SAA: serum amyloid A
TCID_50_: Median tissue culture infective dose
TLRs: Toll-like receptors
