# Supplementary figures and images for: Transcriptome analysis reveals differential immune related genes expression in bovine viral diarrhea virus-2 infected goat peripheral blood mononuclear cells (PBMCs)
Source: BMC Genomics. 2019 Jun 21;20:516. doi: 10.1186/s12864-019-5830-y (PMC6588900; doi:10.1186/s12864-019-5830-y)

Color Key

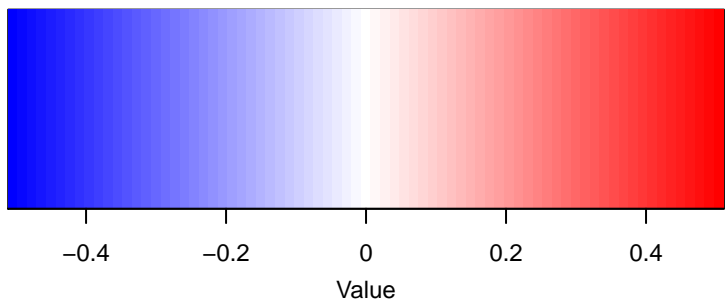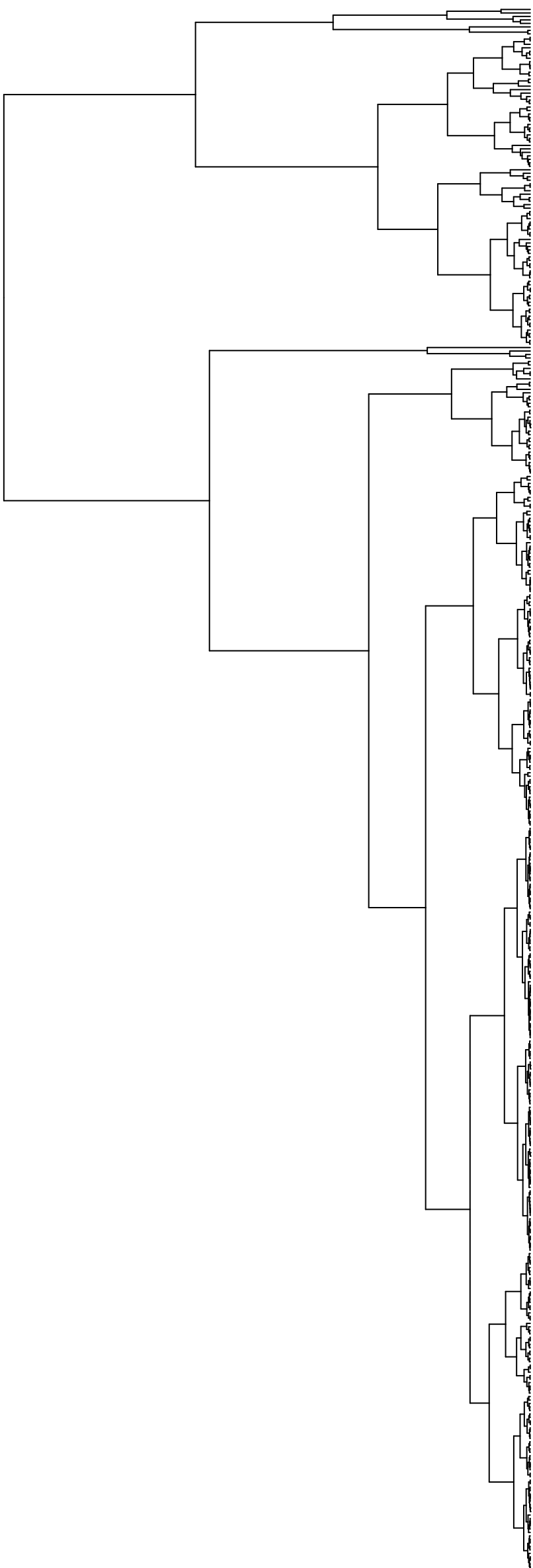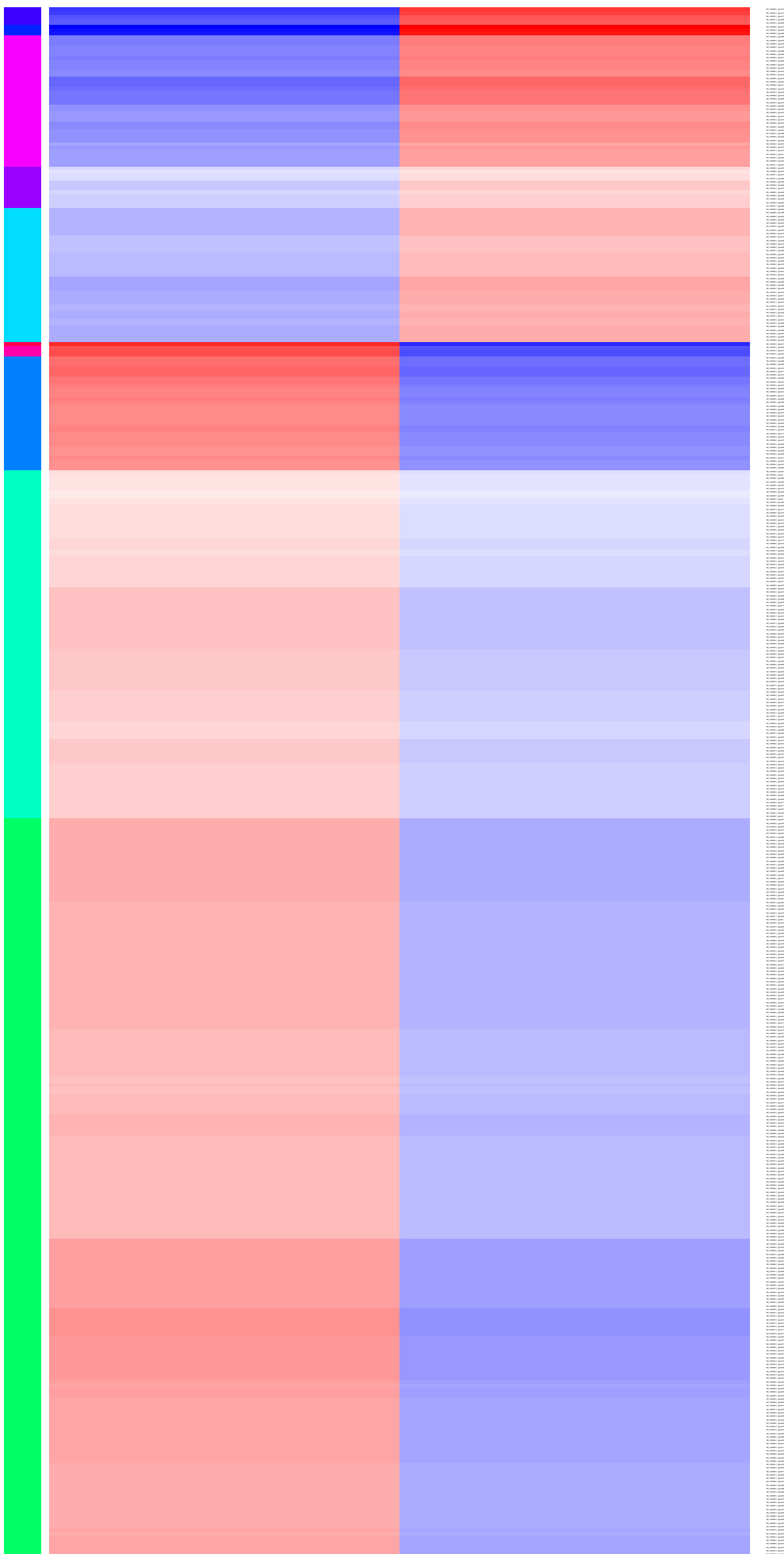

Supplement: Supplementary file 4 — Figure S1. Heat map of the DEGs between mock and BVDV-2 infected samples. Red indicates up-regulation and blue indicates down-regulation. (PDF 47 kb) [file 12864_2019_5830_MOESM4_ESM.pdf]
